# Engineering Doping and Vacancy in a C_3_N_4_ Electrocatalyst with Ni_4_Mo Cocatalyst for Efficient Alkaline Hydrogen Evolution

**DOI:** 10.1021/acsomega.5c10575

**Published:** 2026-01-07

**Authors:** Hsin-An Lin, Sheng-Chang Wang, Jow-Lay Huang, Yu-Min Shen, Wen-Hui Sophia Cheng

**Affiliations:** † 34912 National Cheng Kung University, Department of Materials Science and Engineering, 1 University Road, East District, 701 Tainan, Taiwan; ‡ 34909 Southern Taiwan University of Science and Technology, Department of Mechanical Engineering, 1 Nantai Street, Yongkang District, 710 Tainan, Taiwan; § National Cheng Kung University, Center for Resilience and Intelligence on Sustainable Energy Research (RiSER), 1 University Road, East District, 701 Tainan, Taiwan; ∥ National Cheng Kung University, Center for Quantum Frontiers of Research & Technology (QFort), 1 University Road, East District, 701 Tainan, Taiwan

## Abstract

To meet the growing demand for sustainable hydrogen production, robust and cost-effective electrocatalysts are essential, especially under alkaline conditions. Herein, we report a phosphorus-doped carbon nitride (P–C_3_N_4_) electrocatalyst exhibiting remarkable hydrogen evolution reaction (HER) performance in an alkaline electrolyte. Phosphorus doping was found to promote electronic conductivity and create Lewis acidic active sites, facilitating water dissociation and hydrogen adsorption. Electrochemical measurements revealed a significant reduction in overpotential and Tafel slope upon optimal P-doping (2.0 at%). Further introduction of nitrogen vacancies (NV) and coloading with Ni_4_Mo bimetallic alloy synergistically enhanced the catalytic activity, delivering an overpotential (η_10_) as low as 93 mV at −10 mA·cm^–2^ and a Tafel slope of 88 mV·dec^–1^, without reliance on noble metals. These results underscore the promise of defect-engineered C_3_N_4_ systems as viable catalysts for green hydrogen generation.

## Graphical abstract

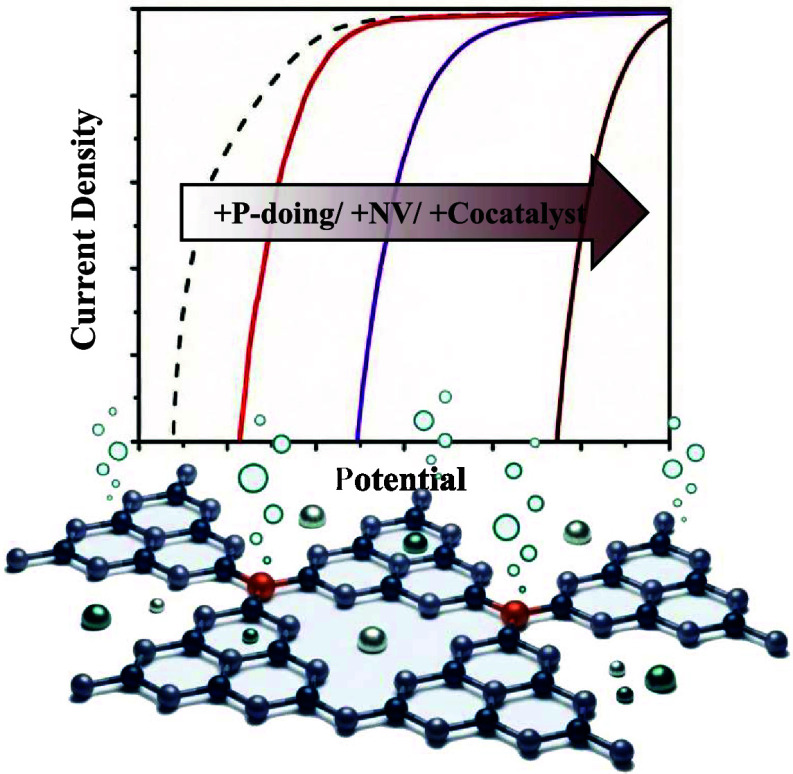

## Introduction

Hydrogen energy has the advantages of high energy density, low cost, high stability, and environmental friendliness. Producing hydrogen gas through water electrolysis using renewable or nuclear power is considered to have the lowest carbon footprint.^1^ This type of hydrogen, termed “green hydrogen,” is gradually becoming a future trend.^2,3^ Currently, acidic systems account for much of the research on electrocatalysis. There is already an academic consensus on the active sites of the catalyst surface, revealing the reaction mechanisms of different types of catalysts and then designing better catalysts. Many nonprecious metal catalysts comparable to Pt have been developed in the laboratory.^4^ However, in the real environment, acidic electrolytes tend to reduce the life of electrolyzers and therefore increase costs.^5,6^ This research aims to develop active, cost-effective, and stable electrocatalysts that can be used in alkaline environments.

From an atomic point of view, hydrogen production from hydrolysis at the cathode involves a hydrogen adsorption reaction (Volmer step) and a hydrogen desorption reaction (Heyrovsky step and Tafel reaction).^7^ Figure 1 shows the reaction equations and schematic diagrams of the two reaction mechanisms in acidic and alkaline electrolytes. For the Volmer step in an acidic environment, the catalyst will directly adsorb hydrogen ions from the electrolyte to form an adsorbed hydrogen atom (H_ad_). In contrast, with an alkaline environment, the Volmer step involves the adsorption of water molecules on the catalyst surface and the breaking of the O–H bond to form adsorbed hydrogen ions (H_ad_). Then, it undergoes the Heyrovsky step or Tafel step to form hydrogen gas. Therefore, HER in an alkaline environment requires additional bond-breaking energy, lowering the reaction rate than in acidic electrolytes. It can be correlated with three factors: (1) the larger the OH^–^ ion is, the slower the mass transfer rate is than that of H^+^; (2) more energy is needed to break the O–H bond in water molecules; and (3) the elements are more oxophilic under the basic environment, leading to the occupation of the active sites by hydroxyl groups.

**Figure 1:**
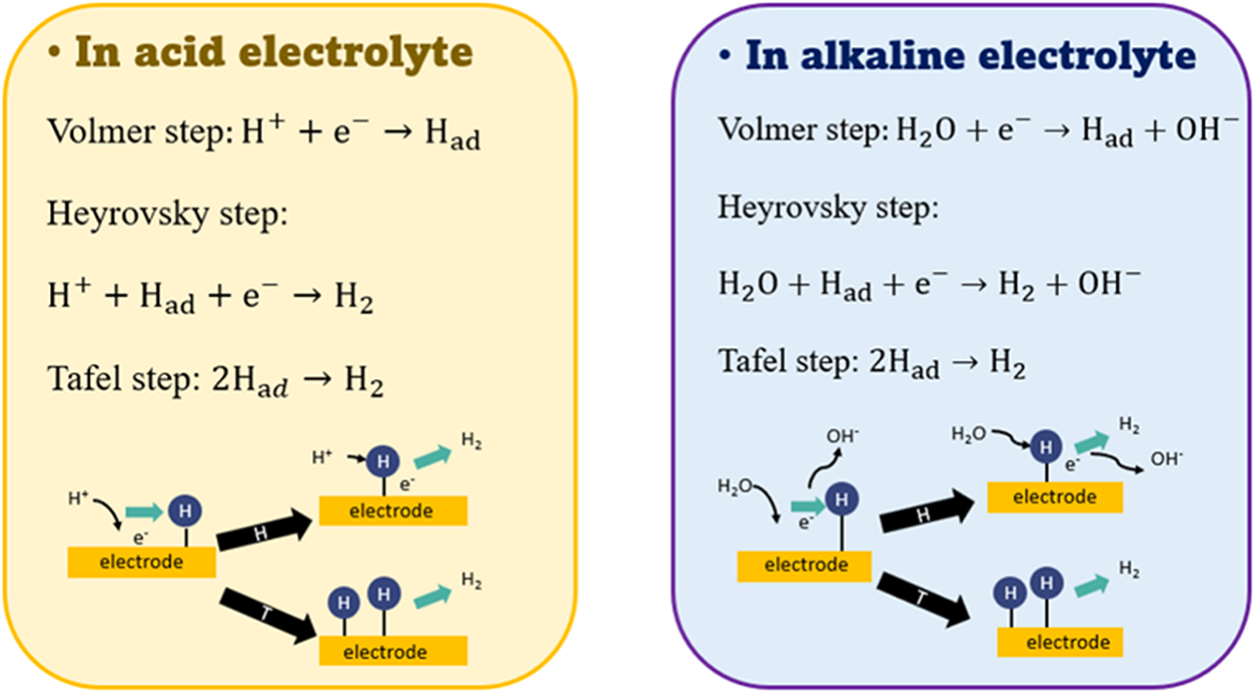
Schematic diagram of the reaction mechanism of hydrogen production from hydrolysis in acidic and alkaline electrolytes.

Carbon nitride (C_3_N_4_), a metal-free polymeric semiconductor, has garnered attention due to its earth-abundant composition, structural stability, and high nitrogen content. The intrinsic nitrogen-rich framework offers potential for hydrogen spillover and catalytic activity. However, its poor intrinsic conductivity and limited active site density constrain HER efficiency.^8,9^ Table S1 summarizes the catalytic activities of different modification strategies for C_3_N_4_ in the alkaline electrolyte. Hyeonuk Choi et al. prepared a 2D/2D heterostructure electrocatalyst composed of C_3_N_4_ and rGO, which promoted better electron transfer due to the strong electric field at the interface between electrodes. It can be regarded as a catalytic active site for redox reactions.^10^ Shiravani et al. proposed an effective strategy to directly synthesize a black-red phosphorus-doped carbon nitride composite (BRP-C_3_N_4_) through one-step thermal treatment. This catalyst exhibited elevated performance for OER and HER. This improvement can be attributed to the chemical structure, which provided efficient active sites and enhanced charge transfer properties.^11^

Han et al. generated functional Cl-doped C_3_N_4_ nanorings with efficient photo-oxidation ability by integrating doping and texture engineering.^12^ Jiang et al. proposed a simple method to directly synthesize C_3_N_4_ and nitrogen-doped graphene (NG) by loading ultrasmall low-content Ir nanoparticles. C_3_N_4_/NG effectively enhanced catalytic kinetics and prevented the aggregation of Ir nanoparticles to help expose active sites.^13^ Shuo Geng et al. optimized the HER catalytic activity of MoS_2_ by S-doping C_3_N_4_ to form a two-dimensional layered heterojunction. The optimized MoS_2_–C_3_N_4_ catalyst exhibited an excellent and durable HER performance with a low overpotential. The better HER activity can be attributed to the higher charge transfer rate, the adjusted electronic structure, and the more exposed active sites.^14^

In addition to its role as an electrocatalyst, C_3_N_4_ also exhibits semiconductor properties, enabling potential applications in photocatalysis. Prior studies have demonstrated improved photocatalytic hydrogen evolution by incorporating phosphorus dopants or nitrogen vacancies to tailor the band structure and carrier dynamics. For instance, Wang et al. reported enhanced light-driven H_2_ production via partial replacement of corner-site carbon atoms with phosphorus, while Liu et al. highlighted the role of nitrogen vacancies in boosting photocatalytic performance under visible light.^15,16^ However, most of these efforts have focused on photochemical or photoelectrochemical conditions. In contrast, our work centers on electrocatalytic HER under alkaline conditions, emphasizing charge transfer engineering.

Due to their excellent efficiency and low cost, Ni–Mo bimetallic alloy electrocatalysts are extensively studied for alkaline HER. The unique properties of this bimetallic catalyst allow it to modify the electronic structure of the material, significantly impacting its electrocatalytic performance. The energy barrier of the Volmer step can be reduced, which promotes the dissociation of water molecules. Therefore, faster reaction kinetics and higher hydrogen production rates can be realized. The material also presents great stability in alkaline conditions. This is vital for practical application. Additionally, researchers are exploring various support materials to improve the dispersion and availability of active sites on the catalyst surface. Recent studies have shown that optimizing the composition and morphology of Ni–Mo bimetallic alloys can further improve their performance.^17–21^

Combining the strategies of heteroatom doping, vacancy engineering, and hybridization with transition-metal-based cocatalysts, we aim to optimize the C_3_N_4_-based electrocatalysts. Herein, we synthesize phosphorus-doped C_3_N_4_ via a wet chemical route followed by controlled thermal treatments to generate NV. Phosphorus doping introduces positively charged P sites within the C_3_N_4_ framework, enhancing electrical conductivity and generating Lewis acidic sites that promote the Volmer step of HER. Complementarily, NV can modulate the electronic structure, create defect-rich regions, and stabilize the adsorbed hydrogen species.^22^ Ni_4_Mo alloy is subsequently integrated through solid-state sintering. The comprehensive characterization of structural, electronic, and electrochemical properties confirms the critical role of P-doping, NV creation, and Ni_4_Mo synergy in optimizing the electrocatalytic HER activity under alkaline operation. More importantly, the catalysts feature a low-cost synthesis process that can be scalable.

## Material and Methods

The fabrication flow of the catalyst synthesis is illustrated in Figure S1. Melamine as the precursor is placed in a porcelain boat and calcinated with a tube furnace at 550 °C for 4 h under Ar gas (heating rate 5 °C/min) to obtain a yellow color C_3_N_4_. The 300 mg of ground C_3_N_4_ is added to water to form a solution. A cup of 0.1 M phosphoric acid solution as the phosphor precursor is prepared. We then add the designed amounts of phosphoric acid solution into the C_3_N_4_ solution and stir for 8 h at 50 °C, with 3.42, 4.56, 5.70, and 6.84 mL corresponding to doping concentrations of 1.5, 2.0, 2.5, and 3.0 at%. After centrifuging and drying steps, the powders are placed in a porcelain boat and calcinated with a tube furnace at 350 °C for 2 h under Ar gas (heating rate 3 °C/min) to obtain a light-yellow color P–C_3_N_4_.

In order to introduce more defects as active sites on the surface, the aforementioned P–C_3_N_4_ is further calcinated at an elevated temperature of 600 °C for 2 h under Ar gas (heating rate 5 °C/min) to obtain P–NV–C_3_N_4_. The solid phase sintering method is applied to sinter the Ni_4_Mo alloy on P–NV–C_3_N_4_ at 400 °C for 1 h under forming gas (heating rate 3 °C/min) to form a black color Ni_4_Mo/P–NV–C_3_N_4_. The Ni_4_Mo/P–C_3_N_4_ is also prepared with the same process.

To perform the three-electrode measurement, the sample is made into a slurry (40 μL Nafion + 800 μL H_2_O + 200 μL alcohol + 5 mg sample), and we applied 5 μL of it on a glassy carbon electrode as the working electrode (WE) with a geometric surface area of 0.07 cm^2^. A saturated calomel electrode is used as the reference electrode (RE) with a standard potential of 0.24 V (*E*_SCE_^0^ = +0.24 V vs SHE), and the counter electrode (CE) is a platinum electrode. The alkaline electrolyte is 1 M KOH. No IR compensation is applied. Considering the influence of pH value, the following formula is used to correlate the voltage.

V(vs.RHE)=V(vs.SCE)+0.24+0.059×pH;pH=14

The overpotential (η_10_) can be obtained through the LSV curve when the current density is −10 mA·cm^–2^. The LSV measurements were performed with a scan rate of 10 mV·s^–1^ at 25 °C. The relationship between the current density and the overpotential under the electrode operating conditions is plotted logarithmically to obtain a straight line, representing the degree to which the electrode potential needs to be changed to achieve a certain current. The plot of the logarithm of the current density and the overpotential is called a Tafel diagram. The unit of Tafel slope is generally mV·dec^–1^, which means the overpotential needed to obtain current changes is ten times.

The results of EIS are presented in the Nyquist plot. The corresponding equivalent circuit diagram in the electrochemical process can be inferred to calculate the relevant component parameters by analyzing and fitting the electrochemical impedance spectroscopy results. The equivalent circuit configuration is usually composed of several resistors and capacitors. *R*_ct_ (charge transfer resistance) is the charge transfer resistance during the redox reaction on the surface of the electrocatalyst. The value is proportional to the charge transfer rate. *C*_dl_ (double-layer capacitance) is the capacitance value of the electric double layer, indicating the amount of charge stored on the surface of the electrocatalyst, and *R*_s_ is the series resistance of the entire system. The EIS in this work is measured as a three-electrode configuration with an applied potential of η_onset_ (obtained from LSV measurement). The frequency range and amplitude are set as 10mHz–1 MHz and 5 mV, respectively. The electrochemical active surface area (ECSA) measurements were conducted in 1 M KOH, and the potential window was selected at the OCV ± 50 mV.

## Results and Discussion

Figure 2 exhibits the proposed structure with phosphorus doping and supporting material characterizations. In order to confirm whether C_3_N_4_ is formed as expected after calcination of melamine, the XRD spectra of the produced products are compared with the JCPDS standard card number No. 87–1526, as shown in Figure 2b. The main signals come from 13° and 27°, representing the (100) crystal plane and the (002) crystal plane, respectively.^10,23^ Comparing the XRD patterns of C_3_N_4_ doped with the P element and pristine C_3_N_4_, it can be found that no new peak is generated. It can be revealed that the doping element does not affect the crystal structure of the material.

**Figure 2:**
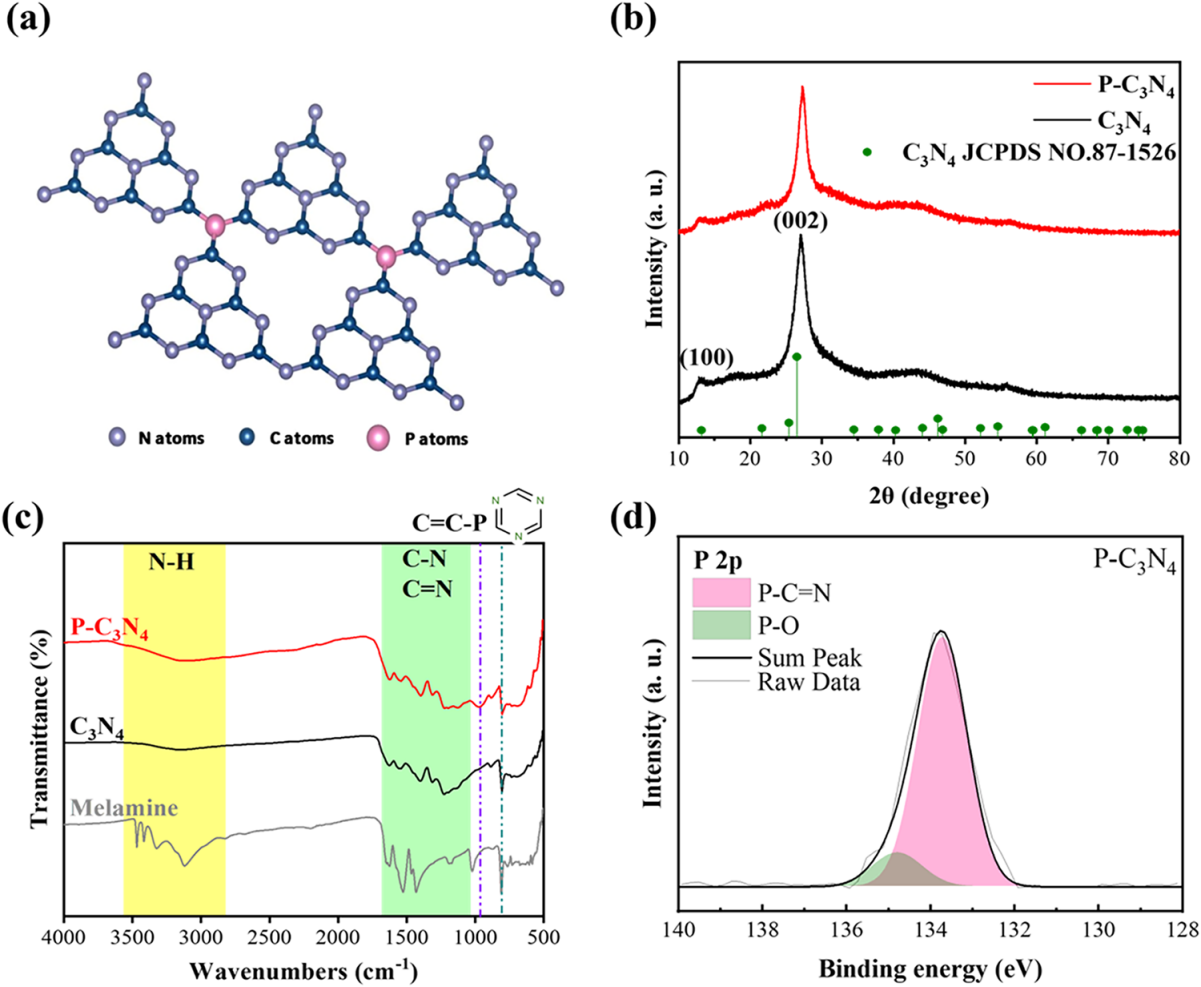
(a) Illustration of the C_3_N_4_ structure with phosphorus doping, (b) XRD spectra of the pristine C_3_N_4_ and P–C_3_N_4_, (c) FTIR spectra of the melamine, pristine C_3_N_4_, and P–C_3_N_4_, and (d) XPS data of P 2p core level for the P–C_3_N_4_ sample.

The absorption peaks corresponding to the transmittance dips in Fourier transform infrared spectroscopy (FTIR) represent the bonding status of the compound, as shown in Figure 2c. The 2800–3560 cm^–1^ band represents N–H bonding (indicated in yellow color), and the 1020–1690 cm^–1^ band represents C–N and C=N bonding (indicated in green color). In addition, there is a signal from the carbon–nitrogen heterocycle at about 810 cm^–1^. It is found that the signals of melamine and C_3_N_4_ are significantly different in both the yellow and green bands. This is because melamine has a single six-membered-ring structure, while the monomer of C_3_N_4_ comprises three six-membered rings. Therefore, it can be determined that melamine has been successfully converted. Comparing the FTIR spectra of P–C_3_N_4_ and pristine C_3_N_4_, similar bonding results in almost identical spectra. The main difference appears at 975 cm^–1^, where P–C_3_N_4_ presents an additional transmittance dip. This dip represents the bonding of P–C=N. In addition to confirming the success of doping, it can also help us to understand the location of phosphorus doping. This conclusion is also consistent with the position indicated by most of the literature.^23,24^

Figures 2d and S2 show the X-ray photoelectron spectroscopy (XPS) data of P 2p for the P–C_3_N_4_ sample. The deconvoluted peaks of P–C=N and P–O can be mainly observed, but the bond between P and N is not detected.^25^ Therefore, it can be inferred that P replaces the position of N in the substituted carbon–nitrogen heterocycle. Through XPS and FTIR, we confirm that for P–C_3_N_4_, phosphorus replaces nitrogen atoms in the heterocyclic structure and binds with the alkenyl carbon. The P at P–C=N exhibits a partial positive charge corresponding to the Lewis acid. Under alkaline conditions, the Lewis acidic P^+^ active sites can effectively adsorb and reduce the water molecules, facilitating the Volmer step in HER. Therefore, the phosphorus can serve as active sites for HER catalytic reactions and can improve the original poor conductivity of C_3_N_4_.^23,26,27^

Electrochemical performance was evaluated using linear sweep voltammetry (LSV) and Tafel analysis. Figure 3a presents an overlay of the LSV curves of pristine C_3_N_4_ and P–C_3_N_4_. It can be found that P–C_3_N_4_ has the smaller overpotential of 770 mV at −10 mA·cm^–2^ while C_3_N_4_ has the worse value, requiring up to 918 mV. Figure 3b shows the Tafel slope. A reduction from 273 mV·dec^–1^ to 200 mV·dec^–1^ can be realized with phosphorus doping. Pristine C_3_N_4_ exhibited a high overpotential and Tafel slope, indicating sluggish HER kinetics. Therefore, it can be inferred that doping the P element into electrocatalyst C_3_N_4_ can effectively improve the electrochemical properties and reduce the applied voltage required for the reaction.

**Figure 3:**
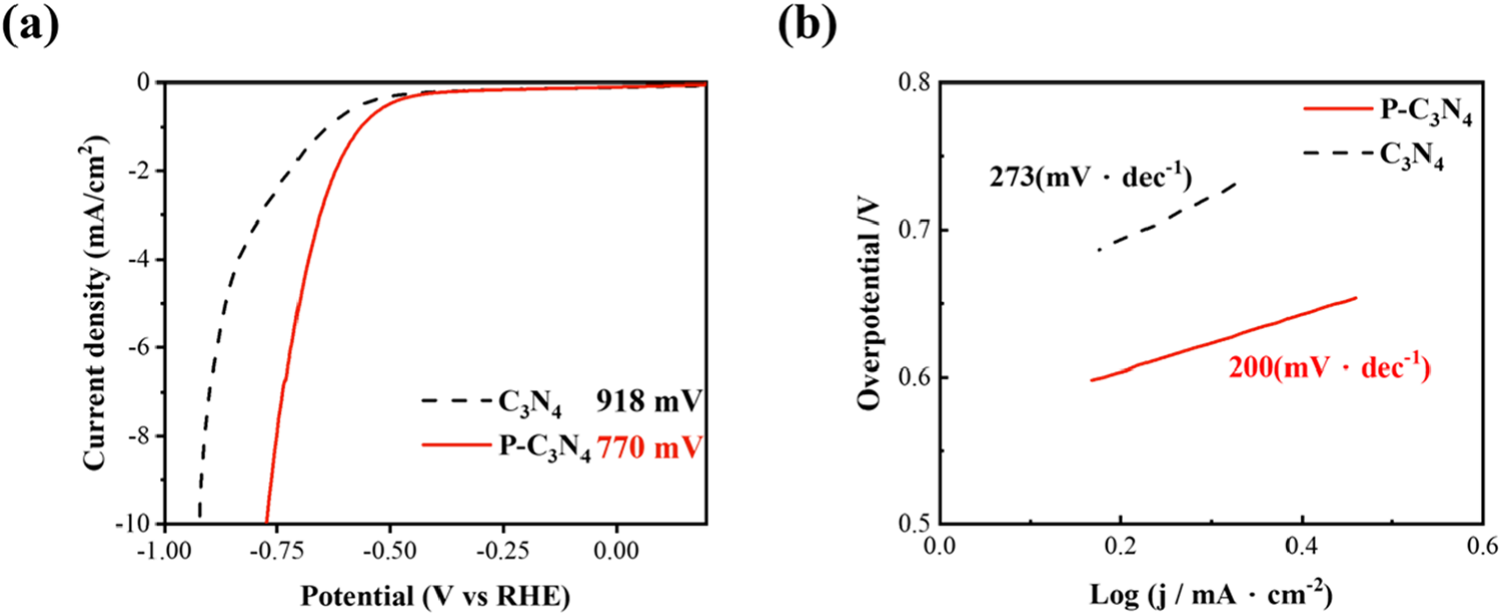
(a) LSV curves of the pristine C_3_N_4_ and P–C_3_N_4_; (b) Tafel slopes of pristine C_3_N_4_ and P–C_3_N_4_.

Quantitative experiments are conducted to determine the best phosphorus doping ratio to optimize the electrochemical properties of P–C_3_N_4_. A range of doping concentrations from 1.5, 2.0, 2.5, to 3.0 at% is chosen, labeled as 1.5P–C_3_N_4_, 2.0P–C_3_N_4_, 2.5P–C_3_N_4_, and 3.0P–C_3_N_4_, respectively. It can be seen from Figure 4 that as the doping amount of phosphorus increases, the overpotential and Tafel slope do not increase monotonically but show the highest value at the doping concentration of 2 at%. By doping 2 at% P, compared with undoped C_3_N_4_, it is found that the overpotential can be reduced from the original 918 to 651 mV, while the Tafel slope is reduced from 273 to 163 mV·dec^–1^.

**Figure 4:**
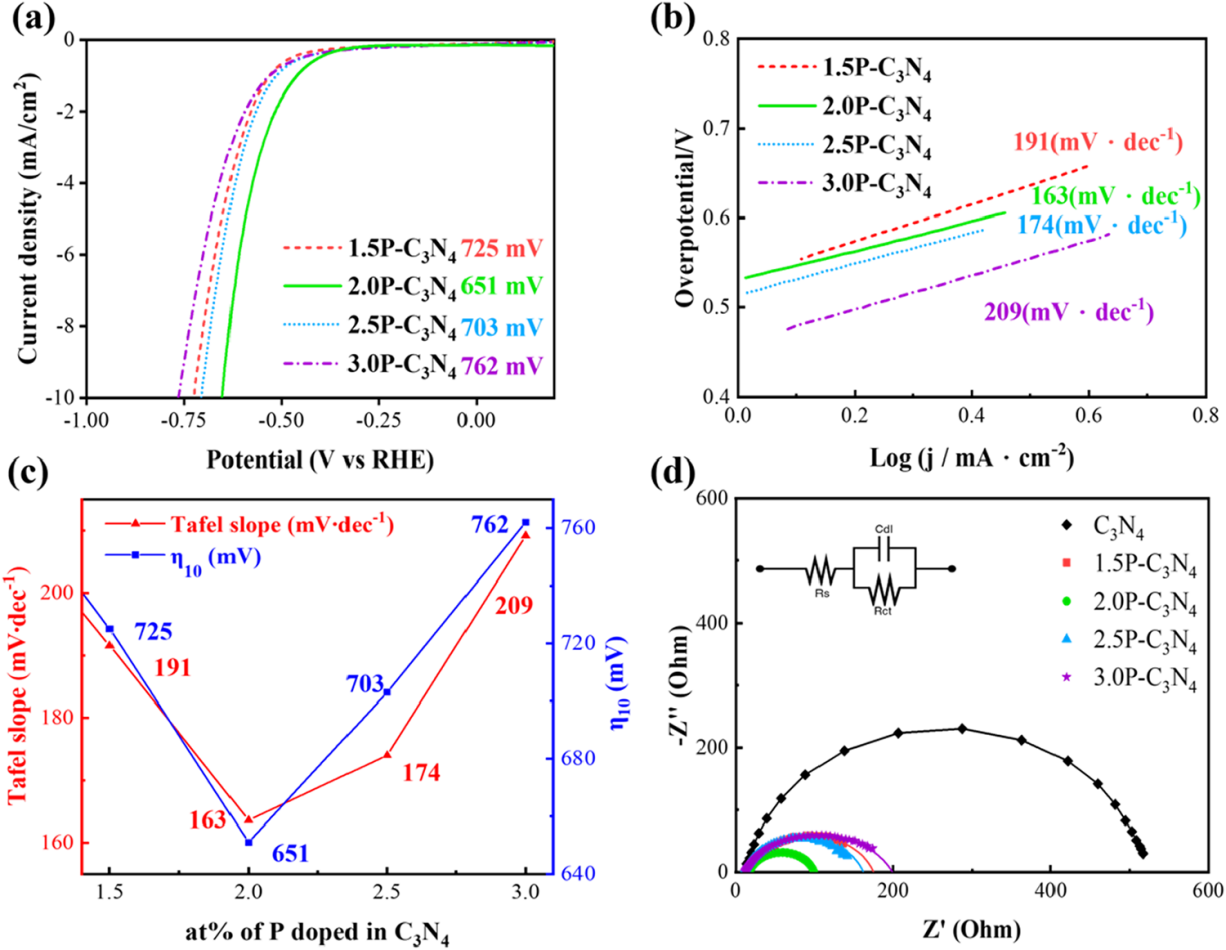
(a) LSV curves of the P–C_3_N_4_ with different doping concentrations, (b) Tafel slopes of the P–C_3_N_4_ with different doping concentrations, (c) correlation of catalytic parameters with different doping concentrations, and (d) Nyquist plots of the pristine C_3_N_4_ and P–C_3_N_4_ with different doping concentrations (the inset shows the equivalent circuit).

To further investigate the charge transfer capability of the catalyst at the interface, the HER dynamics at the electrode–electrolyte interface are analyzed by electrochemical impedance spectroscopy (EIS; tested at η_onset_ vs RHE). The radius of the semicircle in the impedance spectrum is related to the charge transfer resistance of the catalysts. The smaller the radius, the lower the resistance to charge transfer (*R*_ct_) during the HER process. Figure 4d shows the Nyquist plots of pristine C_3_N_4_ and P–C_3_N_4_ with different doping concentrations, and the corresponding equivalent circuit diagrams are drawn in the inset. The fitted components of the EIS response are given in Table S2. The pristine C_3_N_4_ has a larger *R*_ct_ of 527 Ω, which can be attributed to the poor conductivity, hindering the migration of electrons from carbon nitride to adsorbed hydrogen. The result is comparable to the values reported in the literature, as shown in Table S3, which typically range from 500 to 700 Ω depending on the measurement setup.^15,28^

After the addition of P atoms into C_3_N_4_, defect-mediated π conjugation may be effectively induced at the edge of the carbon material, thereby increasing the electron transfer rate. A dramatic reduction of *R*_ct_ to 82 Ω can be achieved with P–C_3_N_4_, which is beneficial for reducing the charge recombination rate. In addition, the *C*_dl_ of P–C_3_N_4_ is much higher, which means that more charges accumulate as the double layer at the catalyst/electrolyte interface. The lowest *R*_ct_ of 2.0P–C_3_N_4_ can correspond to the best charge transfer at the catalytic surface, leading to the lowest overpotential and Tafel slope. According to Figure S2, we found that the peak centers of the P–C=N are 133.76, 133.73, 133.80, and 133.84 eV for 1.5P–C_3_N_4_, 2.0P–C_3_N_4_, 2.5P–C_3_N_4_, and 3.0P–C_3_N_4_, respectively. The results show that as the doping concentration increases, the P site on the catalyst becomes more positively charged. When the doping amount of phosphorus is too high, the positive charge (P^+^) may cause repulsion between the layers. If the doping amount of phosphorus is too low, the overpotential will remain high due to the lack of active sites.

In order to further improve the electrochemical properties and increase the active sites on the surface of 2.0P–C_3_N_4_, secondary calcination at elevated temperature is applied to introduce NV with phosphorus doping. The sample is denoted as 2.0P–NV–C_3_N_4_. With phosphorus doping and incorporation of NV, the overpotential decreases to 595 mV, and the Tafel slope decreases to 140 mV·dec^–1^, as shown in Figure 5a,b. The comparison of Nyquist plots for 2.0P–C_3_N_4_ and 2.0P–NV–C_3_N_4_ is shown in Figure 5c, with the fitted parameters shown in Table S2. It can be observed that with the addition of NV, the *R*_ct_ becomes 73 Ω, which is slightly smaller than the *R*_ct_ of 82 Ω for the 2.0P–C_3_N_4_. It implies that the generation of NV can influence the local electronic structure, improving the interfacial charge transfer. Similarly, the *C*_dl_ of 2.0P–NV–C_3_N_4_ is higher, proving that more charges accumulate at the interface. Therefore, the existence of NV can stabilize the adsorbed hydrogen species, reducing the overpotential required to drive the HER.

**Figure 5:**
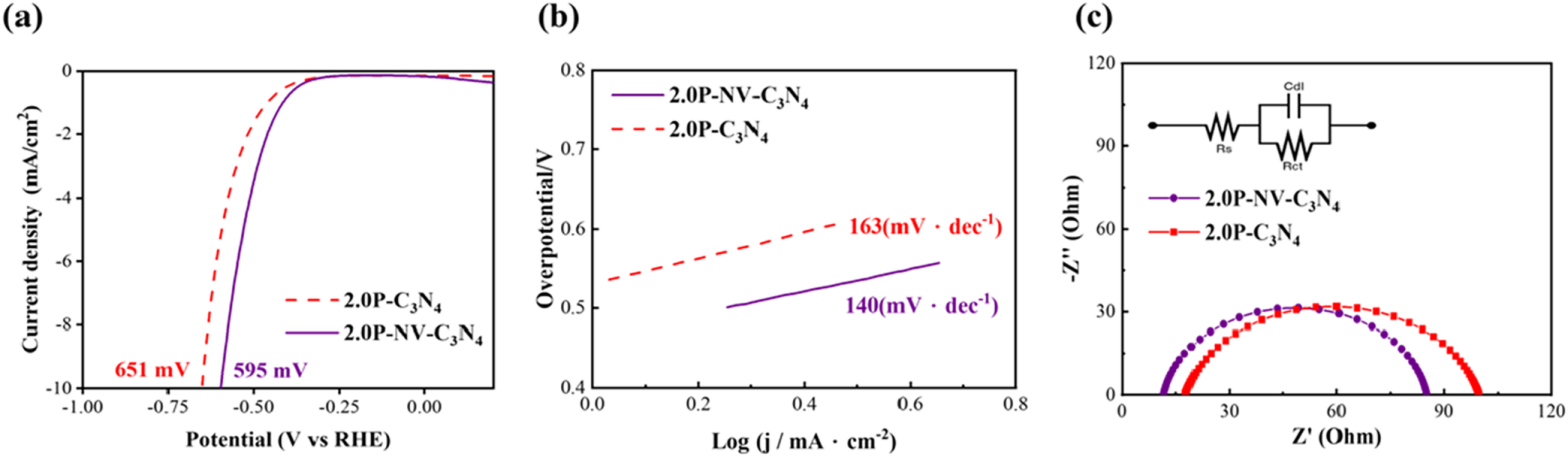
(a) LSV curves of 2.0P–C_3_N_4_ and 2.0P–NV–C_3_N_4_, (b) Tafel slopes of 2.0P–C_3_N_4_ and 2.0P–NV–C_3_N_4_, and (c) Nyquist plots of 2.0P–C_3_N_4_ and 2.0P–NV–C_3_N_4_, with the inset showing the equivalent circuit.

Lastly, we adopt the strategy of increasing active sites with cocatalysts. Ni_4_Mo alloy bimetallic catalysts are reported to have HER activity that can exceed that of Pt.^29^ During alkaline HER, Ni_4_Mo plays an important role in the reaction to conduct H_2_O adsorption and dissociation. In order to clarify the formation of the Ni_4_Mo alloy, we have included a comparison between the experimental XRD pattern of Ni_4_Mo/2.0P–NV–C_3_N_4_ and the corresponding Ni_4_Mo simulation result calculated by Vesta (Figure S3). The matching diffraction peaks at 43.7 and 51.8° are assigned to the (121), (002) planes of Ni_4_Mo, which confirm that the catalyst exhibits alloy characteristics consistent with the expected phase structure.

The structural morphology and composition analysis through SEM/EDS and TEM are shown in Figures S4 and S6 and Table S4. The SEM images show irregular morphology with nanoparticle features around 100 nm. The uniform distribution of the Ni and Mo elements with an atomic ratio around 4:1 can be observed, indicating successful mixing of the C_3_N_4_ and the Ni_4_Mo cocatalysts. The content of the bimetallic cocatalysts is around 13.4 wt %, which was measured by EDS. The TEM results exhibit electron diffraction from C_3_N_4_ and Ni_4_Mo. The presence of Ni and Mo in the catalysts is also confirmed in the XPS data, as shown in Figure S7.^30,31^

Figure S8 shows the XPS data of the N 1s core level for the pristine C_3_N_4_, 2.0P–C_3_N_4_, and Ni_4_Mo/2.0P–NV–C_3_N_4_ sample. The peaks at binding energies of about 399, 400, and 401 eV represent the pyridyl C–N=C, three-coordinate nitrogen-bonded N-(C)_3_, and amino N–H. The C–N=C bonds show the highest content. The ratios of C–N=C to N–(C)_3_ are 3.075 for pristine C_3_N_4_, 3.646 for 2.0P–C_3_N_4_, and 2.98 for the Ni_4_Mo/2.0P–NV–C_3_N_4_ sample, respectively. The lower ratio can be correlated to the formation of more two-coordination NV after high-temperature treatment, which can stabilize the absorbing water molecules under alkaline HER.

In the electrochemical measurement, as shown in Figure 6a,b, we find from the polarization curve of Ni_4_Mo/2.0P–C_3_N_4_ that the overpotential is successfully reduced to 234 mV, and the Tafel slope is also reduced to 95 mV·dec^–1^. It is worth noting that the benefit of applying a nitrogen vacancy is even more evident. The Ni_4_Mo/2.0P–NV–C_3_N_4_ sample shows the lowest overpotential of 93 mV and the lowest Tafel slope of 88 mV·dec^–1^. Compared to 2.0P–NV–C_3_N_4_, the integration of Ni_4_Mo alloy nanoparticles provides an appropriate amount of HER active sites, yielding remarkable catalytic enhancement.

Through the Nyquist plots from EIS, as shown in Figure 6c, we observe the improvement of the charge transfer capability with the Ni_4_Mo cocatalysts. The fitted parameters are listed in Table S2. It is worth noting that the series resistance (*R*_s_) drops dramatically after incorporating the Ni_4_Mo, which can correspond to the improvement of material conductivity. With the synergistic effect of the phosphorus doping, NV, and Ni_4_Mo cocatalysts, the *R*_ct_ is successfully reduced to 16 Ω, contributing to the best HER kinetics of the catalyst. The much larger *C*_dl_ also indicates a high density of electrochemically active sites. A similar trend can be observed from ECSA, as shown in Figures S10 and S11, and Table S5.

**Figure 6:**
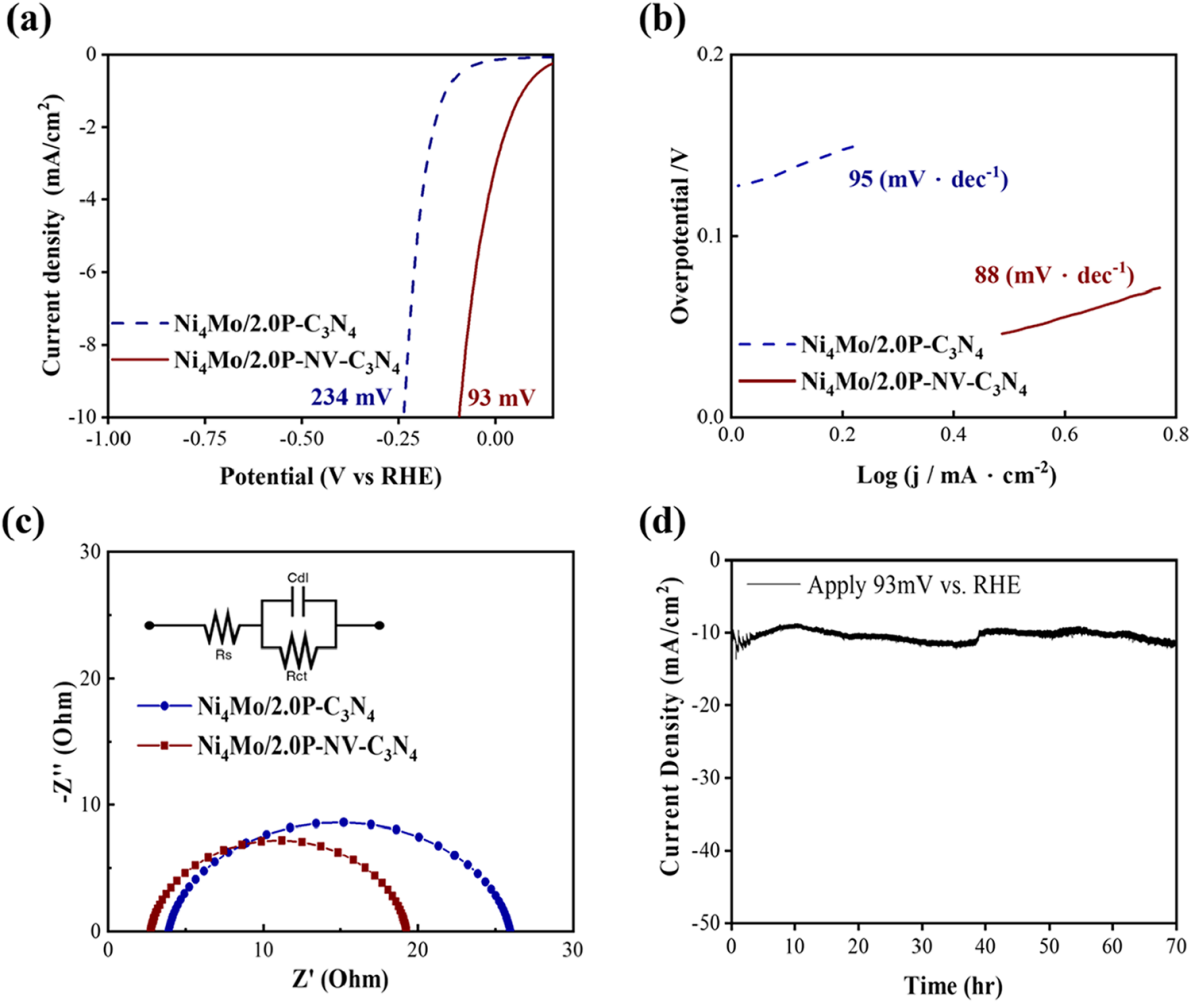
(a) LSV curves of the Ni_4_Mo/2.0P–C_3_N_4_ and Ni_4_Mo/2.0P–NV–C_3_N_4_, (b) Tafel slopes of the Ni_4_Mo/2.0P–C_3_N_4_ and Ni_4_Mo/2.0P–NV–C_3_N_4_, (c) Nyquist plots of the Ni_4_Mo/2.0P–C_3_N_4_ and Ni_4_Mo/2.0P–NV–C_3_N_4_, with inset showing the equivalent circuit, and (d) stability measurement of the Ni_4_Mo/2.0P–NV–C_3_N_4_ electrode at a constant potential of 93 mV vs RHE in 1 M KOH.

The comparison of the catalytic performance at different alkaline concentrations is included in Figure S9. The optimized electrolyte concentration is obtained at 1 M KOH. The viscosity will increase if the KOH concentration is too high, resulting in slower mass transfer. Excessive hydroxyl radicals may also poison the active sites.^32^ If the KOH concentration is too low, it will affect the kinetics of the Volmer step, causing the Tafel slope to increase.^33^

The result of the champion sample Ni_4_Mo/2.0P–NV–C_3_N_4_ is benchmarked against literature-reported C_3_N_4_-based HER catalysts in Table S1, demonstrating superior performance among nonprecious metal systems. To understand the material’s stability during the electrochemical reaction under alkaline conditions, we conduct current measurement at a constant potential of 93 mV vs RHE up to 70 h, as shown in Figure 6d. Negligible degradation is observed, confirming the durability of the catalyst in alkaline conditions. To evaluate the possible influence of metal contamination from the Pt counter electrode, we conducted control LSV measurements using both a Pt wire and a carbon rod as counter electrodes with the same catalyst. As shown in Figure S12, the overpotentials obtained from both setups showed negligible differences, indicating that the use of a Pt wire did not significantly affect the electrochemical results.

## Conclusions

In this work, we report a rationally designed electrocatalyst based on phosphorus-doped C_3_N_4_ with NV and Ni_4_Mo cocatalyst integration, achieving highly efficient catalysis for water splitting. Based on Nyquist plots from EIS, we confirm that optimal phosphorus doping into C_3_N_4_ effectively enhances charge transfer at the interface between the catalyst and electrolyte, thereby improving the kinetics of the HER. Introducing NV in the material can further modulate the electronic structure and reduce the energy barrier. We successfully incorporated the Ni_4_Mo alloy as the cocatalyst of the 2.0P–NV–C_3_N_4_ proposed in this study to improve the conductivity and the active sites, resulting in an extraordinary reduction of Tafel slope and overpotential. This multifunctional modification significantly improves the HER activity under alkaline conditions without the use of noble metals. The proposed strategy offers a scalable and cost-effective approach to engineering advanced metal-free catalysts for sustainable hydrogen production.

## Supplementary Material



## Notes

Fabrication flow; XPS data; XRD pattern; SEM images and EDS data; TEM and SAED analysis; electrocatalytic performance at different concentrations of KOH; ECSA analysis; LSV measurements with different counter electrodes; and summary tables of catalytic activities, EIS fitting performance, and comparison with literature (PDF)
