# Supplementary material for: Engineering Doping and Vacancy in a C3N4 Electrocatalyst with Ni4Mo Cocatalyst for Efficient Alkaline Hydrogen Evolution
Source: ACS Omega. 2026 Jan 7;11(2):3390–7. doi: 10.1021/acsomega.5c10575 (PMC12824718; doi:10.1021/acsomega.5c10575)
Supplement: Supplementary file 1 [file ao5c10575_si_001.pdf]

Supporting Information

Engineering Doping and Vacancy in C_3N_4 Electrocatalyst with Ni_4Mo Cocatalyst for Efficient Alkaline Hydrogen Evolution

Hsin-An Lin[1], Sheng-Chang Wang[2], Jow-Lay Huang[1], Yu-Min Shen[3,], and Wen-Hui (Sophia) Cheng[1,3,4,*]*

[1]National Cheng Kung University, Department of Materials Science and Engineering, 1 University Road East District, 701, Tainan, Taiwan

[2]Southern Taiwan University of Science and Technology, Department of Mechanical Engineering, 1 Nantai Street Yungkang District, 710, Tainan, Taiwan

[3]National Cheng Kung University, Center for Resilience and Intelligence on Sustainable Energy Research (RiSER), 1 University Road East District, 701, Tainan, Taiwan

[4] National Cheng Kung University, Center for Quantum Frontiers of Research & Technology (QFort), 1 University Road East District, 701, Tainan, Taiwan

[*]Email: ymshen0728@outlook.com, wcheng@gs.ncku.edu.tw

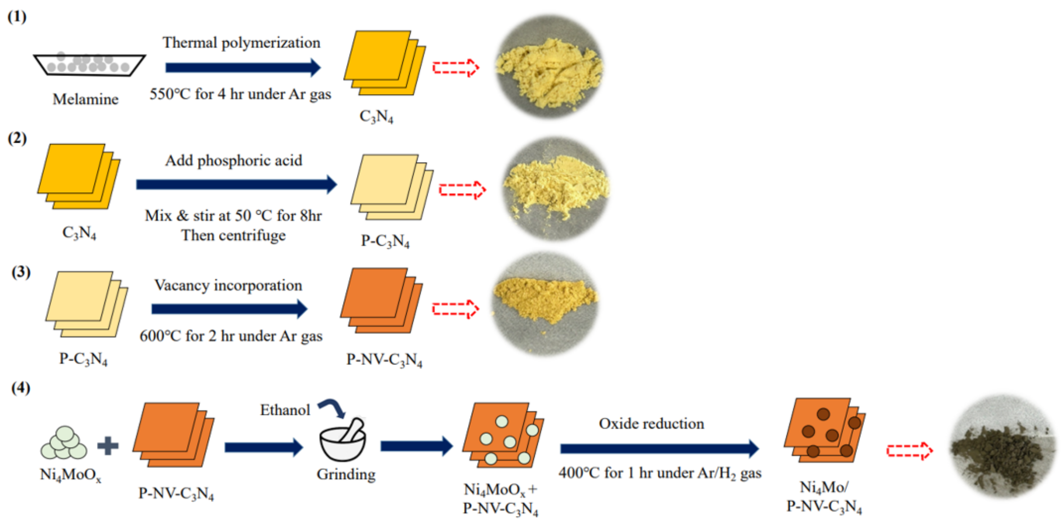

Figure S1. Fabrication flow of (1) C_3N_4 (2) P-C_3N_4 (3) P-NV-C_3N_4 (4) Ni_4Mo/P-NV-C_3N_4

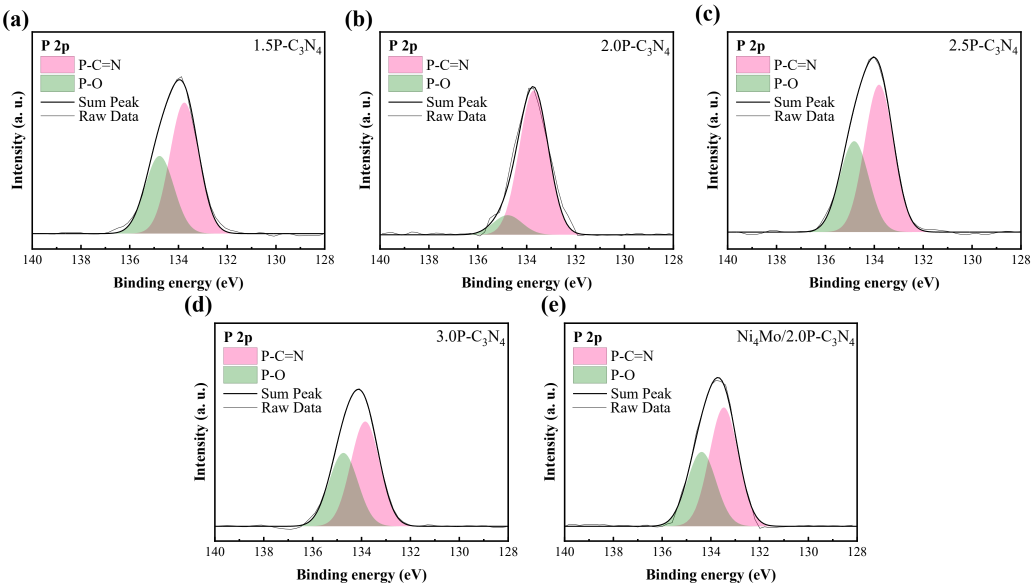

Figure S2. X-ray photoelectron spectra of P 2p core level for the (a) 1.5P-C_3N_4 (b) 2.0P-C_3N_4 (c) 2.5P-C_3N_4 (d) 3.0P-C_3N_4 (e) Ni_4Mo/2.0P-NV-C_3N_4 sample.

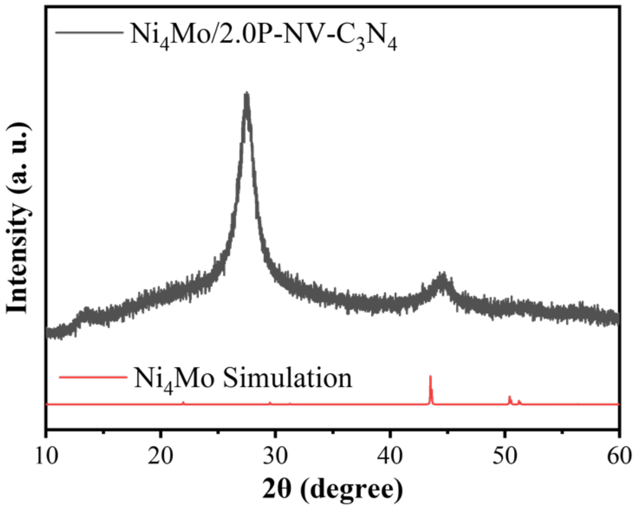

Figure S3. XRD pattern of Ni_4Mo/2.0P-NV-C_3N_4 and the corresponding Ni_4Mo simulation result calculated by Vesta

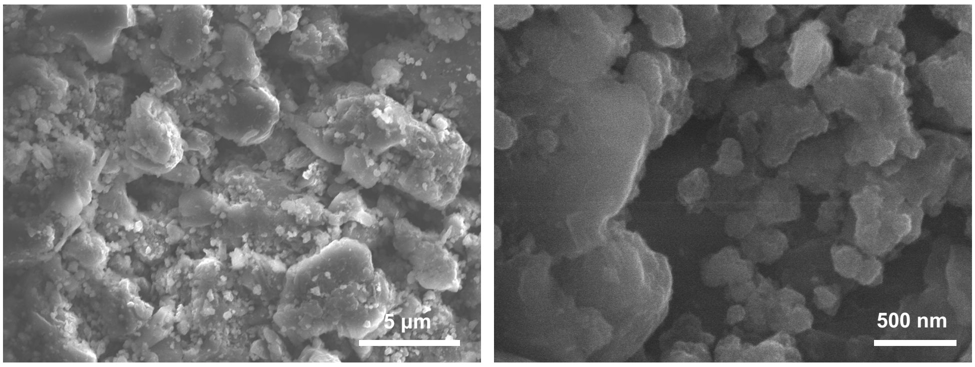

Figure S4. Scanning electron micrographs of the Ni_4Mo/2.0P-NV-C_3N_4 sample.

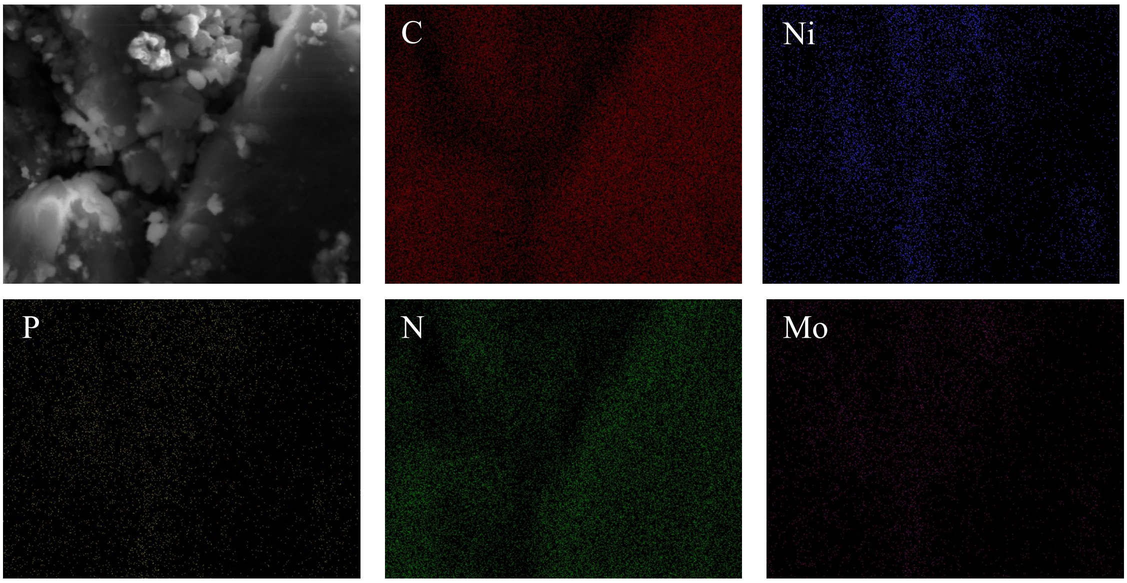

Figure S5. Scanning electron micrographs and energy dispersive spectra (EDS) mappings of elements P, C, N, Ni, and Mo in the $Ni_4Mo/2.0P\text{-}NV\text{-}C_3N_4$ sample.

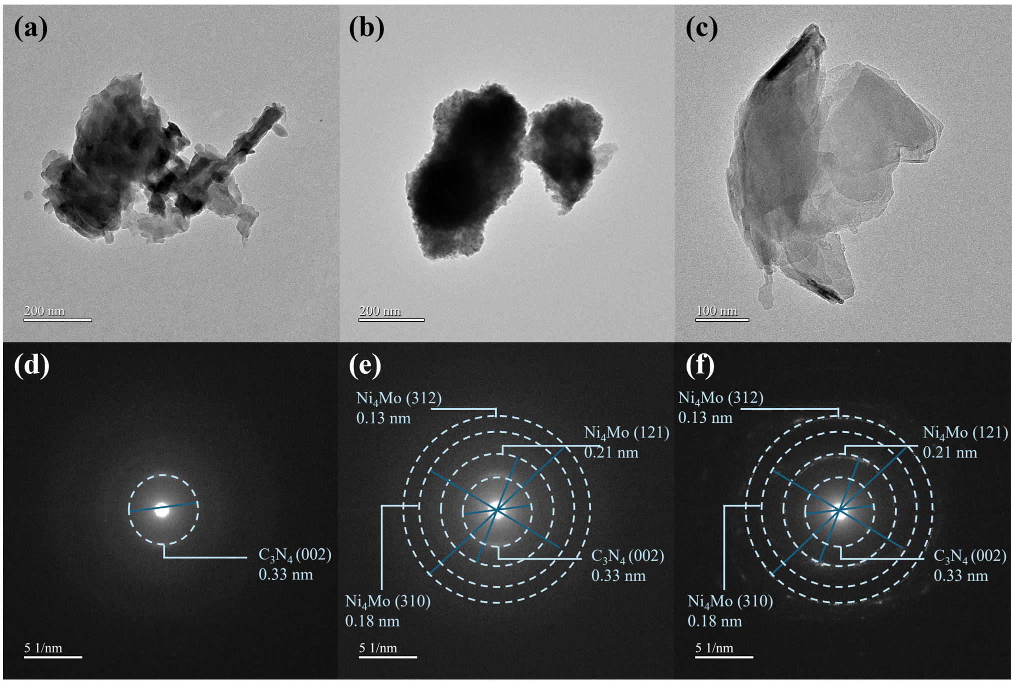

Figure S6. Transmission electron micrographs and SAED of the (a)(d) $2.0P\text{-}NV\text{-}C_3N_4$, (b)(e) $Ni_4Mo/2.0P\text{-}C_3N_4$, (c)(f) $Ni_4Mo/2.0P\text{-}NV\text{-}C_3N_4$ sample

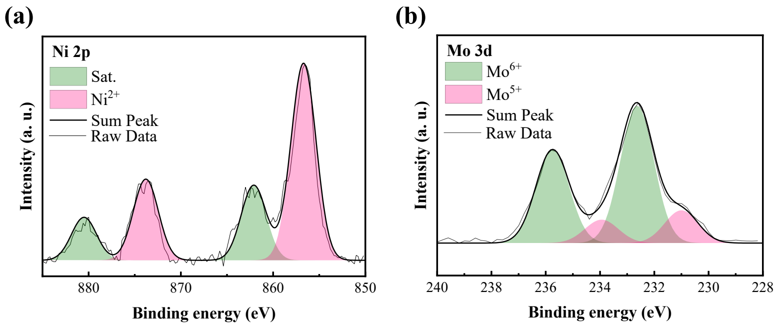

Figure S7. X-ray photoelectron spectra of (a) Ni 2p core level (b) Mo 3d core level for the $Ni_4Mo/2.0P\text{-}NV\text{-}C_3N_4$ sample.

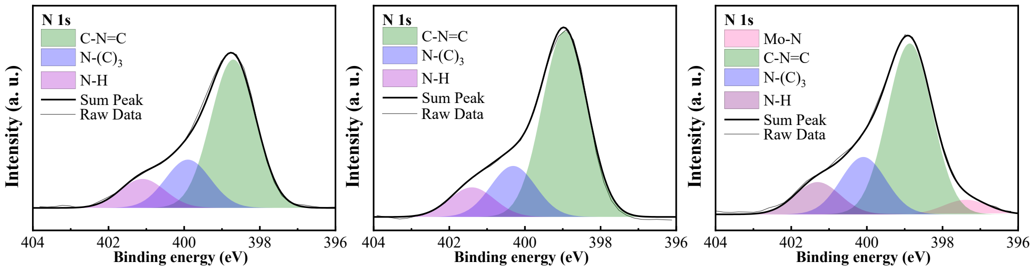

Figure S8. X-ray photoelectron spectra of N 1s core level for the (a) pristine C_3N_4 (b) $2.0P\text{-}C_3N_4$ (c) $Ni_4Mo/2.0P\text{-}NV\text{-}C_3N_4$ sample.

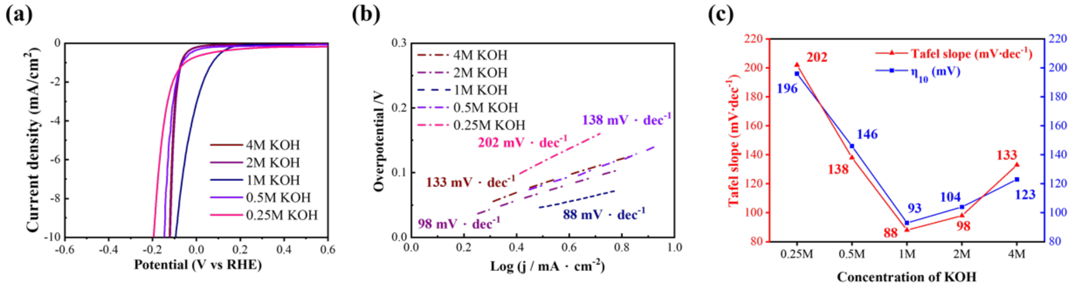

Figure S9. (a) LSV curves of the $Ni_4Mo/2.0P\text{-}NV\text{-}C_3N_4$ at different concentrations of KOH (b) Tafel slopes of the $Ni_4Mo/2.0P\text{-}NV\text{-}C_3N_4$ at different concentrations of KOH (c) Correlation of catalytic parameters with concentrations of KOH. (KOH concentrations are chosen to be 0.25M, 0.5M, 1M, 2M, 4M.)

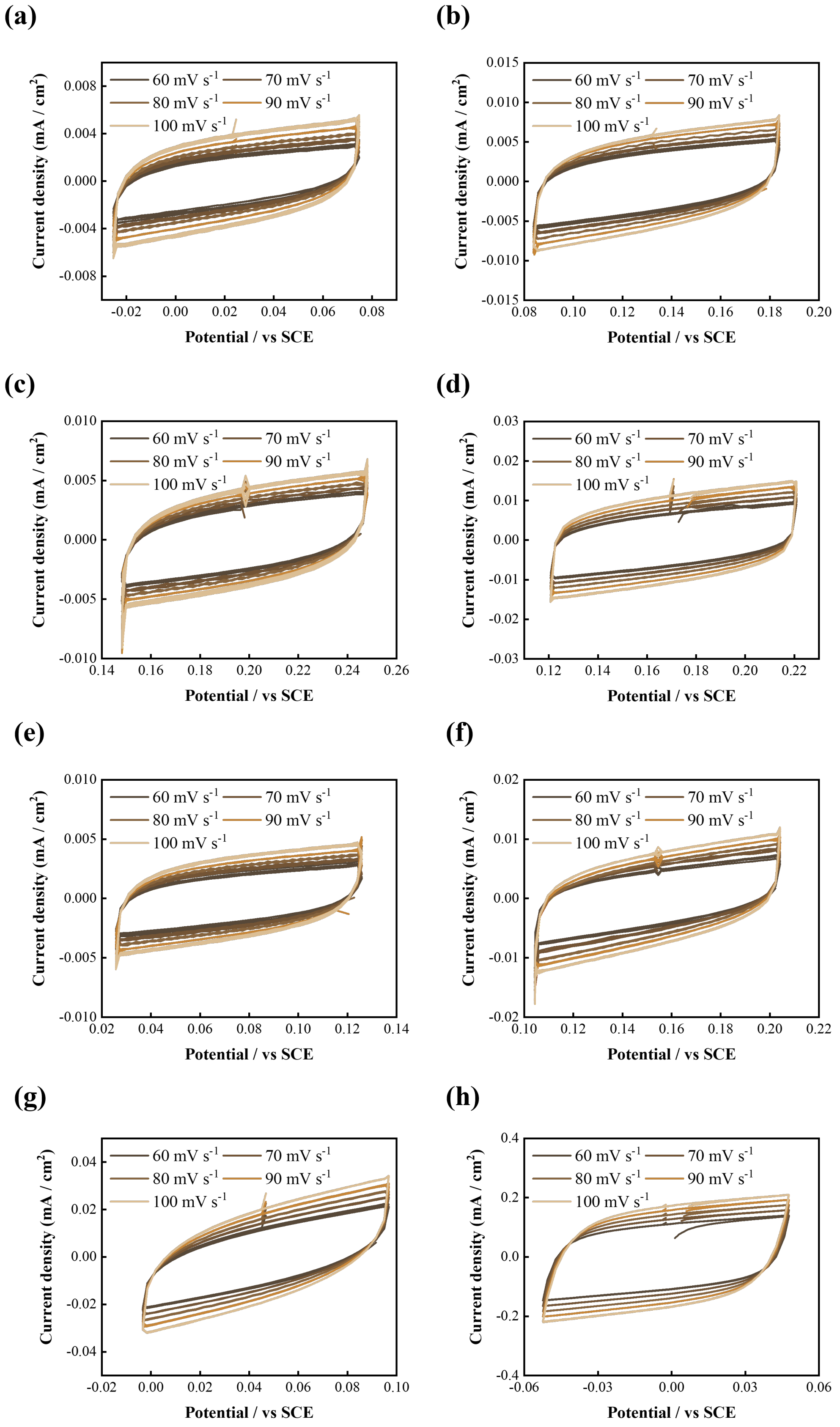

Figure S10. The CV curve of the different samples proposed in this study

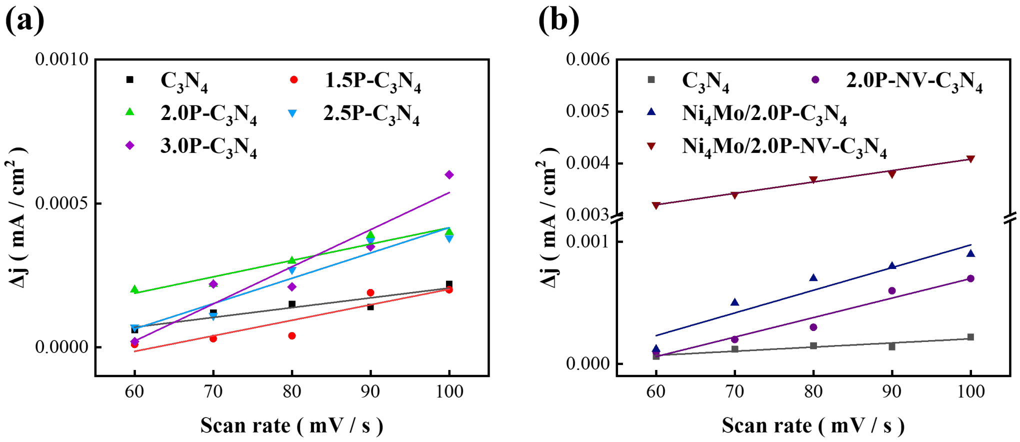

Figure S11. (a) The comparison of ECSA between C_3N_4 and P-C_3N_4 (a) The comparison of ECSA between C_3N_4 and P-C_3N_4 with NV and Ni_4Mo decorated. The ECSA measurements was conducted in 1M KOH, and the potential window was selected at the OCV±50 mV.

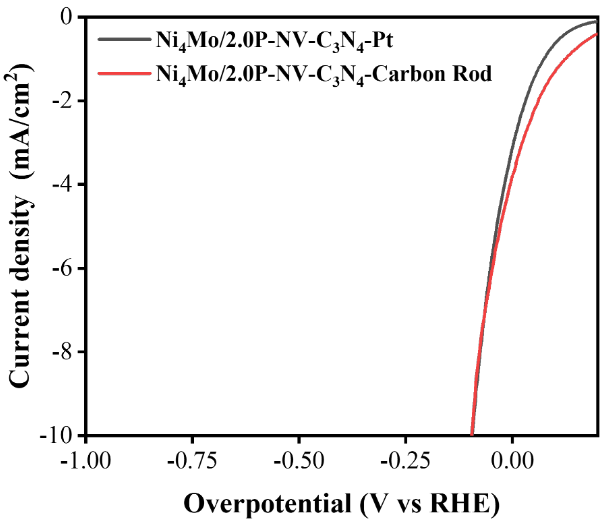

Figure S12. LSV measurements with different counter electrodes

Table S1. A summary of catalytic activities with modified C_3N_4 in alkaline electrolyte

Catalyst	Electrolyte	η_{10} (mV)	Tafel slope (mV dec^{-1})	Year	Ref.
C_3N_4/rGO	1 M KOH	183	164	2022	[10]
BRP-C_3N_4	1 M KOH	115	79	2021	[11]
Cl-doped C_3N_4	1 M KOH	290	55	2019	[12]
Ir/C_3N_4/NG	1 M KOH	22	22	2018	[13]
MoS_2/S-doped C_3N_4	1 M KOH	173	53	2019	[14]
Ni_4Mo/2.0P-NV-C_3N_4	1 M KOH	93	88	2025	This work

Table S2. A summary of catalytic activities and EIS fitting parameters for the different samples proposed in this study

Sample	η_{10} (mV)	Tafel slope (mV/dec)	R_s (Ω)	R_{ct} (Ω)	C_{dl} (μF)
C_3N_4	918	273	17.6	527	1.6
1.5P-C_3N_4	725	191	14.4	146	11.6
2.0P-C_3N_4	651	163	13.7	82	10.2
2.5P-C_3N_4	703	174	9.9	142	18
3.0P-C_3N_4	762	200	11.1	177	13.4
2.0P-NV-C_3N_4	595	140	11.4	73	25.4
Ni_4Mo/2.0P-C_3N_4	234	95	3.9	22	63.6
Ni_4Mo/2.0P-NV-C_3N_4	93	88	2.7	16	71.9

Table S3. The comparison between the Rct value of pristine C_3N_4 in this work and those reported in previous studies.

The R_{ct} of C_3N_4 (Ω)	Electrolyte	Potential (V)	Ref.
527	1M KOH	-0.43V vs RHE	This work
~700	0.5 M Na_2SO_4 +10 vol% TEOA	−0.15 V vs. SCE	[15]
~580	0.5 M $LiClO_4$	-0.4V vs RHE	[28]

Table S4. Composition of elements P, C, N, Ni, and Mo in the Ni_4Mo/2.0P-NV-C_3N_4 sample.

Element	Wt%	At%
C	46.5	56.5
N	37.9	39.5
Ni	9.6	2.4
P	2.2	1.0
Mo	3.8	0.6

Table S5. A summary of ECSA measurements for the different samples proposed in this study.

Sample	C_{dl_ECSA} (μF/cm^2)
C_3N_4	3.4
1.5P-C_3N_4	5.4
2.0P-C_3N_4	5.7
2.5P-C_3N_4	8.8
3.0P-C_3N_4	13
2.0P-NV-C_3N_4	16
Ni_4Mo/2.0P-C_3N_4	18
Ni_4Mo/2.0P-NV-C_3N_4	22

The Calculation of TOF for Ni_4Mo/2.0P-NV-C_3N_4

TOF was calculated through the formula below, which the I stands for current density, n=2 stands for the electron consumption during the HER, F=96485 C/mol, N=the number of active sites.

$$TOF = \frac{I * FE}{n * F * N}$$

The FE was gained through the electrocatalytic H_2 production tested by the GC-BID, the system was applied voltage of η_{10} during the operating time. The FE is about 91%.

The N is equal to $1.77*10^{-8}$ calculated by the weight of catalyst drop casting on the glassy carbon times the weight percentage of phosphorous reported at Table S4.

TOF of Ni_4Mo/2.0P-NV-C_3N_4 is calculated to be 2.66 s^{-1} through the above assumption.
